# Crystal structure of 2-amino-3-ethyl-4,5-di­hydro-1,3-thia­zol-3-ium 3-chloro­benzo­ate

**DOI:** 10.1107/S2056989015008385

**Published:** 2015-05-07

**Authors:** Sara Maira M. Hizam, Bohari M. Yamin

**Affiliations:** a School of Chemical Sciences and Food Technology, Universiti Kebangsaan Malaysia, 43600 Bangi, Selangor D.E., Malaysia

**Keywords:** crystal structure, salt, 3-chloro­benzoate anion, 2-amino-3-ethyl-4,5-di­hydro-1,3-thia­zol-3-ium cation, hydrogen bonding

## Abstract

The title salt, C_5_H_11_N_2_S^+^·C_7_H_4_ClO_2_^−^, comprises a 2-amino-3-ethyl-4,5-di­hydro-1,3-thia­zol-3-ium cation in which the five-membered ring adopts an envelope conformation with the methyl­ene C adjacent to the S atom being the flap, and a planar 3-chloro­benzoate anion (r.m.s. deviation for the 10 non-H atoms = 0.021 Å). The most prominent feature of the crystal packing are N—H⋯O hydrogen bonds whereby the two amine H atoms bridge two carboxyl­ate O atoms resulting in the formation of a centrosymmetric 12-membered {⋯HNH⋯OCO}_2_ synthon involving two cations and two anions. These aggregates are linked by C—H⋯O inter­actions to form a supra­molecular chain along the *a-*axis direction.

## Related literature   

For the crystal structure of a related compound, see: Yamin & Zulkifli (2011 ▸).
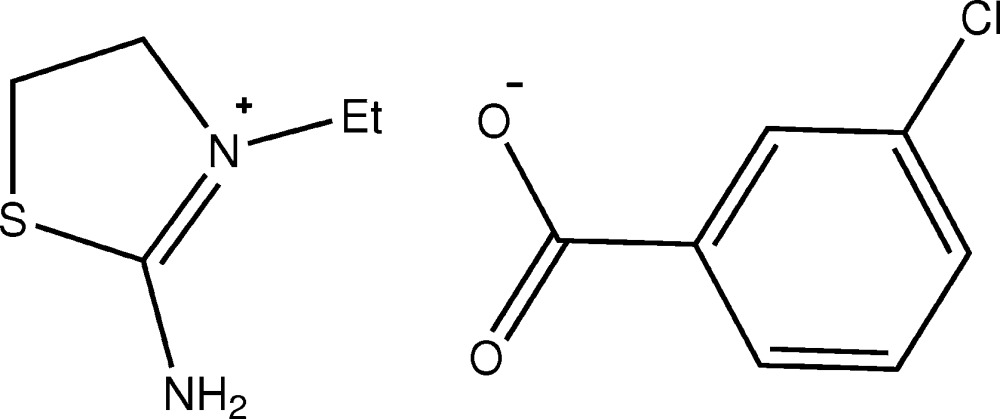


## Experimental   

### Crystal data  

C_5_H_11_N_2_S^+^·C_7_H_4_ClO_2_^−^
*M* *_r_* = 286.77
Triclinic, 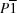

*a* = 7.3376 (7) Å
*b* = 8.7987 (9) Å
*c* = 11.7068 (11) Å
α = 70.728 (3)°
β = 80.269 (3)°
γ = 71.531 (3)°
*V* = 674.95 (11) Å^3^
*Z* = 2
Mo *K*α radiation
μ = 0.43 mm^−1^
*T* = 296 K
0.37 × 0.32 × 0.06 mm

### Data collection  

Bruker SMART APEX CCD area-detector diffractometer
Absorption correction: multi-scan (*SADABS*; Bruker, 2009 ▸) *T*_min_ = 0.856, *T*_max_ = 0.975
16295 measured reflections
3430 independent reflections
1957 reflections with *I* > 2σ(*I*)
*R*_int_ = 0.064

### Refinement  

*R*[*F* ^2^ > 2σ(*F* ^2^)] = 0.063
*wR*(*F* ^2^) = 0.142
*S* = 1.02
3430 reflections
171 parameters
2 restraints
H atoms treated by a mixture of independent and constrained refinement
Δρ_max_ = 0.31 e Å^−3^
Δρ_min_ = −0.26 e Å^−3^

### 

Data collection: *SMART* (Bruker, 2009 ▸); cell refinement: *SAINT* (Bruker, 2009 ▸); data reduction: *SAINT*; program(s) used to solve structure: *SHELXS97* (Sheldrick, 2008 ▸); program(s) used to refine structure: *SHELXL97* (Sheldrick, 2008 ▸); molecular graphics: *SHELXTL* (Sheldrick, 2008 ▸); software used to prepare material for publication: *SHELXTL* and *PLATON* (Spek, 2009 ▸).

## Supplementary Material

Crystal structure: contains datablock(s) global, I. DOI: 10.1107/S2056989015008385/tk5367sup1.cif

Structure factors: contains datablock(s) I. DOI: 10.1107/S2056989015008385/tk5367Isup2.hkl

Supporting information file. DOI: 10.1107/S2056989015008385/tk5367Isup3.cml

**Fig. 1.**
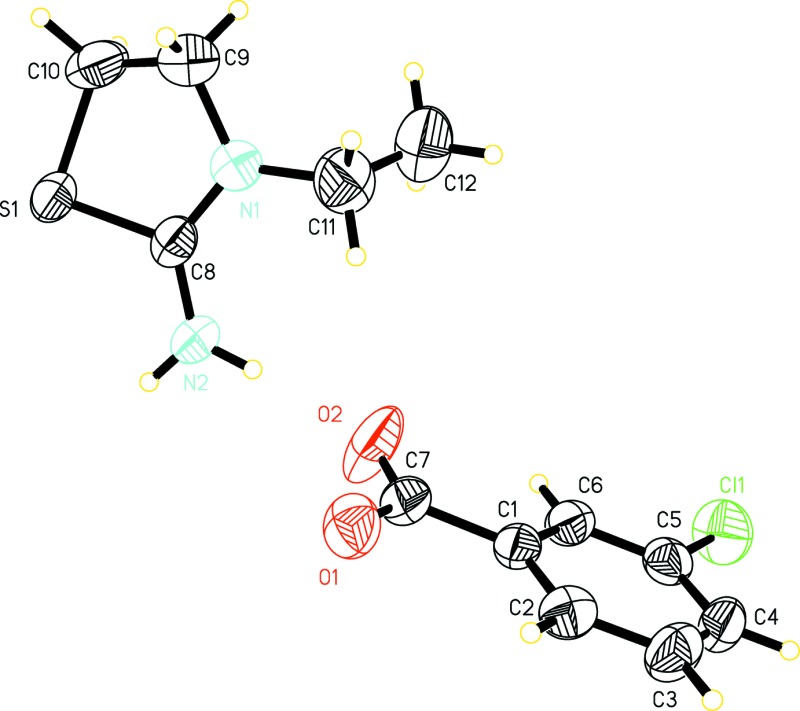
The molecular structure of the title salt with displacement ellipsoids drawn at 50% probability level.

**Fig. 2.**
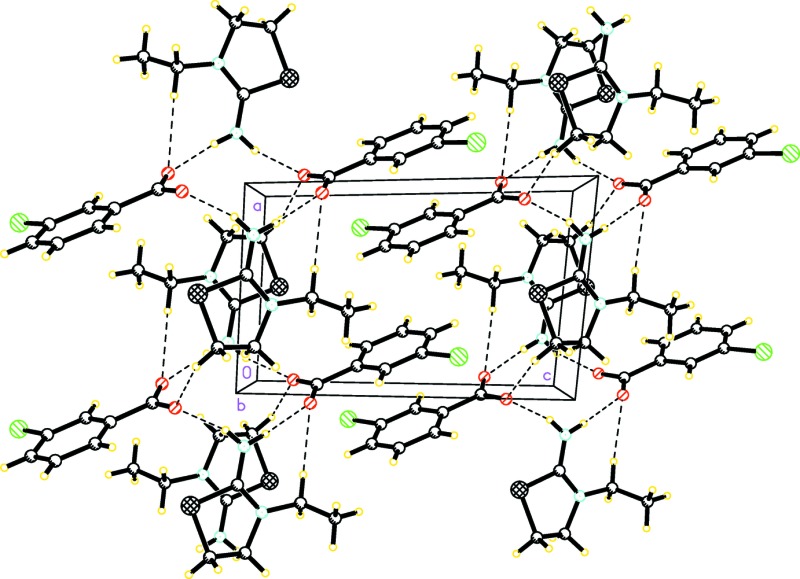
A view of the crystal packing of the title salt viewed down b axis. The dashed lines indicate hydrogen bonds.

. DOI: 10.1107/S2056989015008385/tk5367fig1.tif
The mol­ecular structure of the title salt with displacement ellipsoids drawn at 50% probability level.

b . DOI: 10.1107/S2056989015008385/tk5367fig2.tif
A view of the crystal packing of the title salt viewed down *b* axis. The dashed lines indicate hydrogen bonds.

CCDC reference: 1062249

Additional supporting information:  crystallographic information; 3D view; checkCIF report

## Figures and Tables

**Table 1:** Hydrogen-bond geometry (, )

*D*H*A*	*D*H	H*A*	*D* *A*	*D*H*A*
N2H2*B*O1^i^	0.87(2)	1.89(2)	2.730(3)	164(2)
N2H2*C*O2	0.86(2)	1.83(2)	2.680(3)	169(2)
C10H10*B*O1^ii^	0.97	2.46	3.297(4)	145

Symmetry codes: (i) 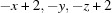; (ii) 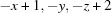.

## supplementary crystallographic information

### S1. Comment

It was reported previously that 3-nitro-4-chlorobenzoyl isothiocyanate reacted with piperidine to give 2,2,6,6-tetramethyl-4-oxopiperidin-1-ium 4-chloro-3-nitrobenzoate (Yamin & Zulkifli, 2011). Similarly, in this study, the reaction of 3-chlorobenzoyl isothiocyanate with 2-ethylaminoethanol also gave an unexpected product, i.e. the title salt, 3-ethylthiazoliden-3-ium-2-amine 3-chlorobenzoate (Fig. 1). The chlorobenzoate Cl1/(C1—C7)/O1/O2 anion is planar with maximum deviation of 0.018 (3) Å for the C3 atom from the least squares plane. The thiazoliden ring S1/N1/C8/C9/C10 is tilted with maximum deviation of 0.159 (3) Å for C10 atom from the least squares plane. The N1—C8 bond length of 1.320 (3) Å indicates the ring nitrogen atom N1 is protonated. In the crystal structure, the molecules are linked by intermolecular hydrogen bonds N2—H2B···O1, C2—H10B···O1, N2—H2C···O2 and C11—H11B···O2 (symmetry codes as in Table 1) to form a one-dimensional chain along the *a* axis (Fig. 2). A weak π.,.π interaction with the distance between (C1—C6) centroids of 3.534 () Å (2 - *x*, -1 - *y* ,3 - *z*) was observed.

### S2. Experimental

An acetone solution (20 ml) of 2-(ethylamino)ethanol (0.01 mol, 0.8914 g m) was added into a two-necked round-bottomed flask containing an equimolar amount of 3-chlorobenzoylisothiocyanate (0.01 mol). The mixture was refluxed for about 3 h, filtered and left to evaporate at room temperature. The filtrate gave colourless crystals after 2 days of evaporation (yield 86.02%, m.pt: 368.2–369.5 K).

### S3. Refinement

H atoms were positioned geometrically with C—H = 0.93–0.97 Å and constrained to ride on their parent atoms with *U*_iso_(H)= 1.2*U*_eq_(CH and CH_2_) and 1.5*U*_eq_(CH_3_). The H atoms on the nitrogen were refined isotropically and with N—H = 0.86±0.01 Å.

### Crystal data

**Table:**

C_5_H_11_N_2_S^+^·C_7_H_4_ClO_2_^−^	*Z* = 2
*M**_r_* = 286.77	*F*(000) = 300
Triclinic, *P*1	*D*_x_ = 1.411 Mg m^−^^3^
Hall symbol: -P 1	Mo *K*α radiation, λ = 0.71073 Å
*a* = 7.3376 (7) Å	Cell parameters from 6990 reflections
*b* = 8.7987 (9) Å	θ = 2.9–28.6°
*c* = 11.7068 (11) Å	µ = 0.43 mm^−^^1^
α = 70.728 (3)°	*T* = 296 K
β = 80.269 (3)°	Slab, colourless
γ = 71.531 (3)°	0.37 × 0.32 × 0.06 mm
*V* = 674.95 (11) Å^3^	

### Data collection

**Table:**

Bruker SMART APEX CCD area-detector diffractometer	3430 independent reflections
Radiation source: fine-focus sealed tube	1957 reflections with *I* > 2σ(*I*)
Graphite monochromator	*R*_int_ = 0.064
Detector resolution: 83.66 pixels mm^-1^	θ_max_ = 28.6°, θ_min_ = 2.9°
ω scan	*h* = −9→9
Absorption correction: multi-scan (*SADABS*; Bruker, 2009)	*k* = −11→11
*T*_min_ = 0.856, *T*_max_ = 0.975	*l* = −15→15
16295 measured reflections	

### Refinement

**Table:**

Refinement on *F*^2^	Primary atom site location: structure-invariant direct methods
Least-squares matrix: full	Secondary atom site location: difference Fourier map
*R*[*F*^2^ > 2σ(*F*^2^)] = 0.063	Hydrogen site location: inferred from neighbouring sites
*wR*(*F*^2^) = 0.142	H atoms treated by a mixture of independent and constrained refinement
*S* = 1.02	*w* = 1/[σ^2^(*F*_o_^2^) + (0.0715*P*)^2^] where *P* = (*F*_o_^2^ + 2*F*_c_^2^)/3
3430 reflections	(Δ/σ)_max_ < 0.001
171 parameters	Δρ_max_ = 0.31 e Å^−^^3^
2 restraints	Δρ_min_ = −0.26 e Å^−^^3^

### Special details

**Table:**

Geometry. All e.s.d.'s (except the e.s.d. in the dihedral angle between two l.s. planes) are estimated using the full covariance matrix. The cell e.s.d.'s are taken into account individually in the estimation of e.s.d.'s in distances, angles and torsion angles; correlations between e.s.d.'s in cell parameters are only used when they are defined by crystal symmetry. An approximate (isotropic) treatment of cell e.s.d.'s is used for estimating e.s.d.'s involving l.s. planes.
Refinement. Refinement of *F*^2^ against ALL reflections. The weighted *R*-factor *wR* and goodness of fit *S* are based on *F*^2^, conventional *R*-factors *R* are based on *F*, with *F* set to zero for negative *F*^2^. The threshold expression of *F*^2^ > σ(*F*^2^) is used only for calculating *R*-factors(gt) *etc*. and is not relevant to the choice of reflections for refinement. *R*-factors based on *F*^2^ are statistically about twice as large as those based on *F*, and *R*- factors based on ALL data will be even larger.

### Fractional atomic coordinates and isotropic or equivalent isotropic displacement parameters (Å^2^)

**Table:**

	*x*	*y*	*z*	*U*_iso_*/*U*_eq_	
Cl1	1.18120 (11)	−0.25267 (10)	1.66658 (6)	0.0724 (3)	
S1	0.48369 (9)	0.37155 (8)	0.90259 (5)	0.0493 (2)	
O1	1.0531 (3)	−0.2998 (2)	1.16212 (16)	0.0570 (5)	
O2	0.9540 (3)	−0.0771 (3)	1.22844 (19)	0.0855 (7)	
N1	0.4326 (3)	0.1591 (3)	1.10804 (19)	0.0523 (6)	
N2	0.7549 (3)	0.1491 (3)	1.0431 (2)	0.0514 (6)	
H2B	0.835 (3)	0.189 (3)	0.9867 (19)	0.064 (9)*	
H2C	0.810 (3)	0.068 (2)	1.1024 (17)	0.059 (8)*	
C1	1.1263 (3)	−0.3301 (3)	1.3595 (2)	0.0383 (5)	
C2	1.2154 (3)	−0.4996 (3)	1.3822 (2)	0.0459 (6)	
H2A	1.2237	−0.5512	1.3230	0.055*	
C3	1.2921 (4)	−0.5927 (3)	1.4918 (2)	0.0545 (7)	
H3A	1.3509	−0.7072	1.5065	0.065*	
C4	1.2829 (4)	−0.5184 (3)	1.5798 (2)	0.0523 (7)	
H4A	1.3353	−0.5812	1.6537	0.063*	
C5	1.1949 (3)	−0.3501 (3)	1.5564 (2)	0.0441 (6)	
C6	1.1164 (3)	−0.2547 (3)	1.4475 (2)	0.0416 (6)	
H6A	1.0571	−0.1405	1.4335	0.050*	
C7	1.0367 (3)	−0.2280 (3)	1.2403 (2)	0.0468 (6)	
C8	0.5708 (3)	0.2094 (3)	1.0315 (2)	0.0406 (6)	
C9	0.2384 (4)	0.2374 (4)	1.0666 (3)	0.0713 (9)	
H9A	0.1472	0.2595	1.1336	0.086*	
H9B	0.2010	0.1635	1.0351	0.086*	
C10	0.2390 (3)	0.3967 (4)	0.9701 (3)	0.0609 (8)	
H10A	0.1996	0.4898	1.0043	0.073*	
H10B	0.1511	0.4184	0.9096	0.073*	
C11	0.4633 (5)	0.0131 (4)	1.2191 (3)	0.0694 (9)	
H11A	0.3912	−0.0612	1.2173	0.083*	
H11B	0.5988	−0.0486	1.2200	0.083*	
C12	0.4029 (5)	0.0639 (4)	1.3300 (3)	0.0829 (10)	
H12A	0.4251	−0.0338	1.3995	0.124*	
H12B	0.2683	0.1232	1.3303	0.124*	
H12C	0.4758	0.1357	1.3330	0.124*	

### Atomic displacement parameters (Å^2^)

**Table:**

	*U*^11^	*U*^22^	*U*^33^	*U*^12^	*U*^13^	*U*^23^
Cl1	0.0822 (6)	0.0905 (6)	0.0555 (5)	−0.0246 (5)	−0.0093 (4)	−0.0333 (4)
S1	0.0453 (4)	0.0562 (4)	0.0395 (4)	−0.0020 (3)	−0.0132 (3)	−0.0117 (3)
O1	0.0605 (11)	0.0701 (13)	0.0473 (10)	−0.0239 (9)	−0.0116 (9)	−0.0172 (10)
O2	0.1099 (18)	0.0587 (14)	0.0682 (14)	0.0216 (12)	−0.0489 (13)	−0.0156 (10)
N1	0.0438 (12)	0.0556 (14)	0.0474 (12)	−0.0072 (10)	−0.0040 (10)	−0.0085 (10)
N2	0.0411 (13)	0.0547 (15)	0.0449 (13)	0.0008 (11)	−0.0117 (11)	−0.0065 (11)
C1	0.0318 (12)	0.0416 (14)	0.0396 (13)	−0.0133 (10)	−0.0043 (10)	−0.0060 (10)
C2	0.0431 (14)	0.0437 (15)	0.0527 (15)	−0.0128 (11)	−0.0046 (12)	−0.0154 (12)
C3	0.0549 (16)	0.0369 (15)	0.0627 (18)	−0.0070 (12)	−0.0120 (14)	−0.0050 (13)
C4	0.0476 (15)	0.0547 (18)	0.0445 (14)	−0.0119 (13)	−0.0136 (12)	0.0011 (13)
C5	0.0371 (13)	0.0566 (16)	0.0417 (14)	−0.0189 (12)	−0.0008 (11)	−0.0138 (12)
C6	0.0366 (13)	0.0378 (13)	0.0473 (14)	−0.0087 (10)	−0.0045 (11)	−0.0096 (11)
C7	0.0361 (13)	0.0573 (18)	0.0436 (14)	−0.0125 (12)	−0.0091 (11)	−0.0080 (13)
C8	0.0463 (15)	0.0370 (13)	0.0376 (13)	−0.0010 (11)	−0.0101 (11)	−0.0167 (11)
C9	0.0454 (17)	0.095 (2)	0.0659 (19)	−0.0181 (16)	−0.0046 (14)	−0.0148 (18)
C10	0.0399 (15)	0.0683 (19)	0.0718 (19)	−0.0033 (13)	−0.0153 (14)	−0.0231 (16)
C11	0.074 (2)	0.0483 (17)	0.068 (2)	−0.0075 (15)	−0.0001 (16)	−0.0059 (15)
C12	0.100 (3)	0.075 (2)	0.063 (2)	−0.0175 (19)	−0.0161 (19)	−0.0080 (18)

### Geometric parameters (Å, º)

**Table:**

Cl1—C5	1.743 (3)	C3—H3A	0.9300
S1—C8	1.746 (2)	C4—C5	1.368 (4)
S1—C10	1.811 (3)	C4—H4A	0.9300
O1—C7	1.245 (3)	C5—C6	1.377 (3)
O2—C7	1.243 (3)	C6—H6A	0.9300
N1—C8	1.320 (3)	C9—C10	1.483 (4)
N1—C9	1.460 (3)	C9—H9A	0.9700
N1—C11	1.485 (3)	C9—H9B	0.9700
N2—C8	1.298 (3)	C10—H10A	0.9700
N2—H2B	0.866 (10)	C10—H10B	0.9700
N2—H2C	0.862 (10)	C11—C12	1.465 (4)
C1—C6	1.379 (3)	C11—H11A	0.9700
C1—C2	1.379 (3)	C11—H11B	0.9700
C1—C7	1.514 (3)	C12—H12A	0.9600
C2—C3	1.376 (3)	C12—H12B	0.9600
C2—H2A	0.9300	C12—H12C	0.9600
C3—C4	1.373 (4)		
			
C8—S1—C10	90.90 (12)	N2—C8—N1	126.9 (2)
C8—N1—C9	115.4 (2)	N2—C8—S1	120.1 (2)
C8—N1—C11	125.1 (2)	N1—C8—S1	112.98 (17)
C9—N1—C11	118.6 (2)	N1—C9—C10	107.9 (2)
C8—N2—H2B	120.1 (19)	N1—C9—H9A	110.1
C8—N2—H2C	126.2 (18)	C10—C9—H9A	110.1
H2B—N2—H2C	114 (3)	N1—C9—H9B	110.1
C6—C1—C2	119.3 (2)	C10—C9—H9B	110.1
C6—C1—C7	120.2 (2)	H9A—C9—H9B	108.4
C2—C1—C7	120.5 (2)	C9—C10—S1	106.45 (18)
C3—C2—C1	120.3 (2)	C9—C10—H10A	110.4
C3—C2—H2A	119.8	S1—C10—H10A	110.4
C1—C2—H2A	119.8	C9—C10—H10B	110.4
C4—C3—C2	120.7 (2)	S1—C10—H10B	110.4
C4—C3—H3A	119.7	H10A—C10—H10B	108.6
C2—C3—H3A	119.7	C12—C11—N1	112.1 (3)
C5—C4—C3	118.6 (2)	C12—C11—H11A	109.2
C5—C4—H4A	120.7	N1—C11—H11A	109.2
C3—C4—H4A	120.7	C12—C11—H11B	109.2
C4—C5—C6	121.6 (2)	N1—C11—H11B	109.2
C4—C5—Cl1	119.55 (19)	H11A—C11—H11B	107.9
C6—C5—Cl1	118.8 (2)	C11—C12—H12A	109.5
C5—C6—C1	119.5 (2)	C11—C12—H12B	109.5
C5—C6—H6A	120.3	H12A—C12—H12B	109.5
C1—C6—H6A	120.3	C11—C12—H12C	109.5
O2—C7—O1	125.3 (2)	H12A—C12—H12C	109.5
O2—C7—C1	116.6 (2)	H12B—C12—H12C	109.5
O1—C7—C1	118.1 (2)		
			
C6—C1—C2—C3	0.5 (4)	C2—C1—C7—O1	−3.0 (3)
C7—C1—C2—C3	−178.3 (2)	C9—N1—C8—N2	−174.0 (3)
C1—C2—C3—C4	−0.6 (4)	C11—N1—C8—N2	−5.0 (4)
C2—C3—C4—C5	0.3 (4)	C9—N1—C8—S1	4.8 (3)
C3—C4—C5—C6	0.0 (4)	C11—N1—C8—S1	173.7 (2)
C3—C4—C5—Cl1	179.99 (19)	C10—S1—C8—N2	−171.3 (2)
C4—C5—C6—C1	−0.1 (4)	C10—S1—C8—N1	9.8 (2)
Cl1—C5—C6—C1	179.95 (18)	C8—N1—C9—C10	−20.9 (4)
C2—C1—C6—C5	−0.2 (3)	C11—N1—C9—C10	169.4 (3)
C7—C1—C6—C5	178.6 (2)	N1—C9—C10—S1	26.1 (3)
C6—C1—C7—O2	−1.0 (3)	C8—S1—C10—C9	−20.7 (2)
C2—C1—C7—O2	177.8 (2)	C8—N1—C11—C12	112.8 (3)
C6—C1—C7—O1	178.2 (2)	C9—N1—C11—C12	−78.6 (4)

### Hydrogen-bond geometry (Å, º)

**Table:**

*D*—H···*A*	*D*—H	H···*A*	*D*···*A*	*D*—H···*A*
N2—H2*B*···O1^i^	0.87 (2)	1.89 (2)	2.730 (3)	164 (2)
N2—H2*C*···O2	0.86 (2)	1.83 (2)	2.680 (3)	169 (2)
C10—H10*B*···O1^ii^	0.97	2.46	3.297 (4)	145

Symmetry codes: (i) −*x*+2, −*y*, −*z*+2; (ii) −*x*+1, −*y*, −*z*+2.

## References

[bb1] Bruker (2009). *SADABS*, *SMART* and *SAINT*. Bruker AXS Inc., Madison, Wisconsin, USA.

[bb2] Sheldrick, G. M. (2008). *Acta Cryst.* A**64**, 112–122. 10.1107/S0108767307043930 18156677

[bb3] Spek, A. L. (2009). *Acta Cryst.* D**65**, 148–155. 10.1107/S090744490804362X PMC2631630 19171970

[bb4] Yamin, B. M. & Zulkifli, N. Z. (2011). *Acta Cryst.* E**67**, o1920. 10.1107/S1600536811025074 PMC3212310 22090967

