# Supplementary figures and images for: Crystal structure of 2-amino-3-ethyl-4,5-di­hydro-1,3-thia­zol-3-ium 3-chloro­benzo­ate
Source: Acta Crystallogr E Crystallogr Commun. 2015 May 7;71(Pt 6):o378. doi: 10.1107/S2056989015008385 (PMC4459344; doi:10.1107/S2056989015008385)

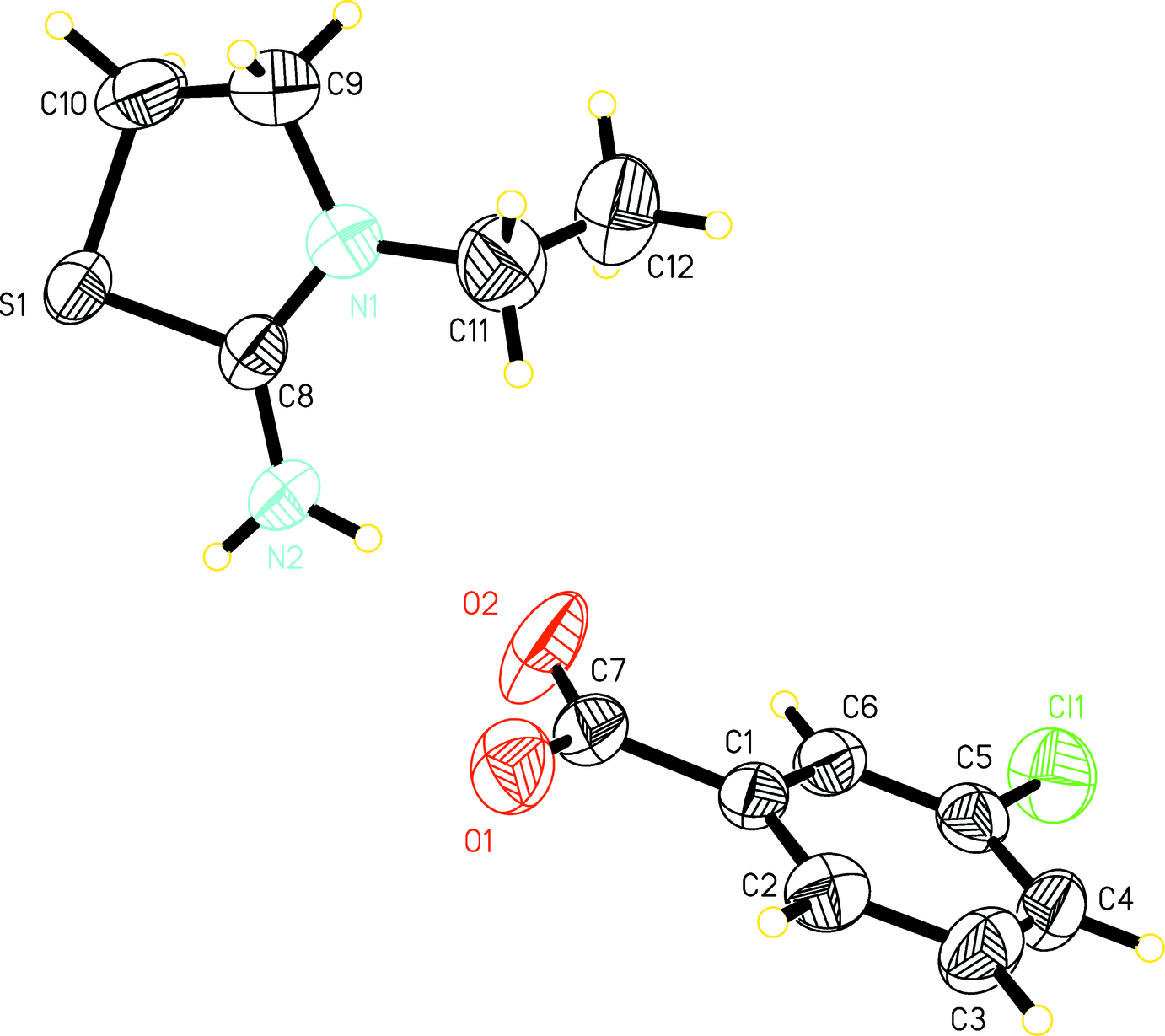

Supplement: Supplementary file 4 [file e-71-0o378-fig1.tif]

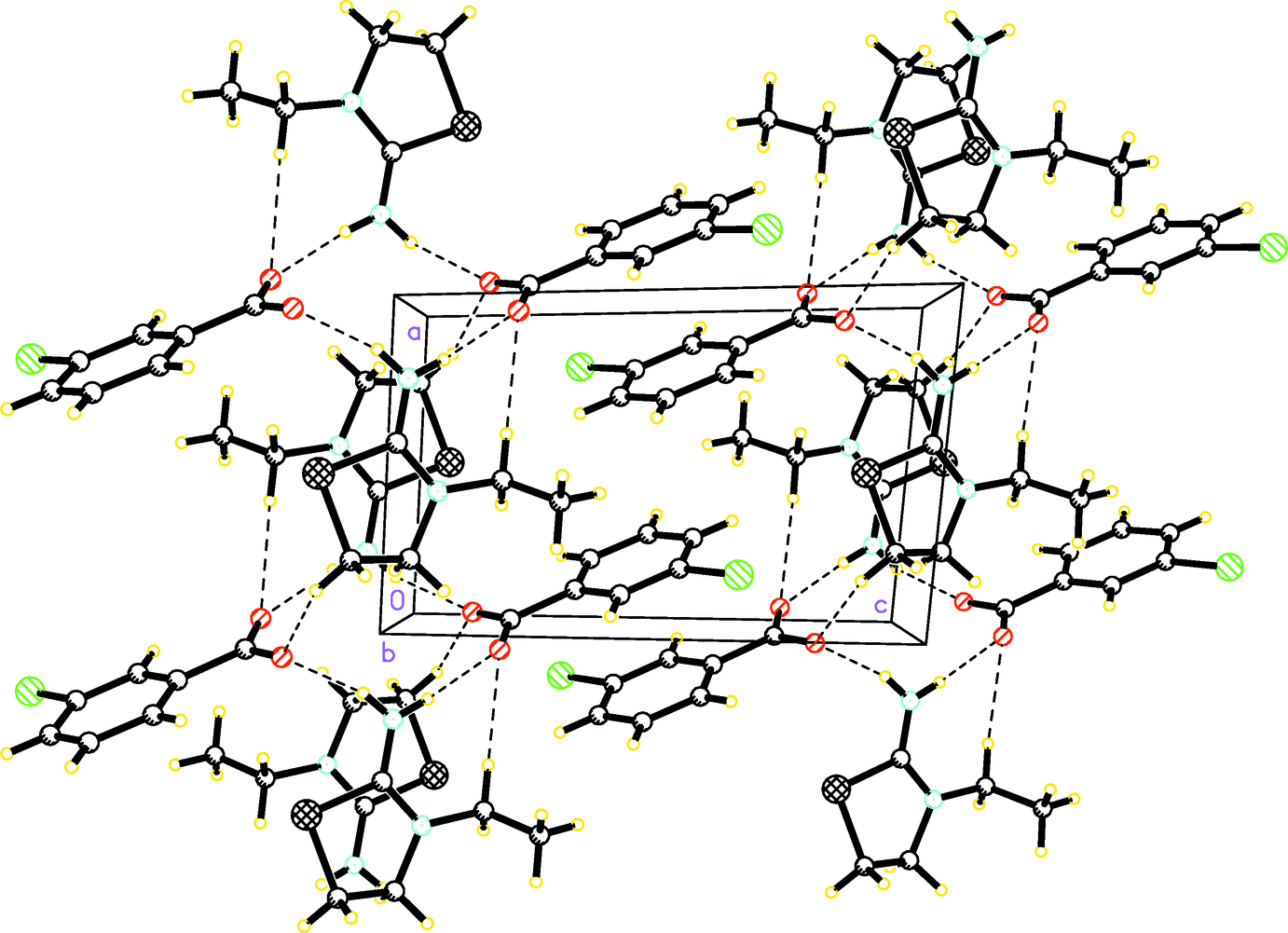

Supplement: Supplementary file 5 [file e-71-0o378-fig2.tif]
